# Anticipating and addressing event-specific alcohol consumption among adolescents

**DOI:** 10.1186/s12889-016-3355-8

**Published:** 2016-07-29

**Authors:** Simone Pettigrew, Nicole Biagioni, Michelle I. Jongenelis

**Affiliations:** School of Psychology and Speech Pathology, Curtin University, GPO Box U1987, Perth, WA 6845 Australia

**Keywords:** Celebrations, Schoolies, Young drinkers, Harm reduction, Qualitative

## Abstract

**Background:**

Various specific events and celebrations are associated with excessive alcohol consumption and related harms. End-of-school celebrations such as Schoolies in Australia are of particular concern given high levels of documented harm among underage and young drinkers. The present study investigated high school students’ expectations of their Schoolies celebrations to inform future interventions to reduce adverse outcomes among members of this vulnerable group and other young people involved in similar rites of passage.

**Methods:**

A link to an online survey was distributed via high schools and Schoolies-related websites. The survey included qualitative questions that invited respondents to discuss (i) aspects of Schoolies they were looking forward to most and least and (ii) their perceptions of the likely consequences if they refrained from consuming alcohol during the event. In total, 435 students provided responses.

**Results:**

Respondents discussed the role of Schoolies in marking their transition to adulthood. Their comments revealed a cross-temporal focus indicating that Schoolies is simultaneously symbolic of the past, present, and future. Through its ability to enhance social interaction, alcohol was perceived to have a vital role in realising the potential of this event to signify and facilitate this temporal progression.

**Conclusions:**

Results suggest interventions that treat Schoolies as an isolated event that occurs in specific locations may fail to appreciate the extent to which these events transcend time for those involved. Instead, harm reduction is likely to involve a reconceptualisation of the event among both participants and authority figures to facilitate the provision of alternative pastimes to drinking during Schoolies that yield similar social benefits.

## Background

As occurs in many countries, Australian youth experience an extended rite of passage to adulthood that involves the years immediately prior to and after the attainment of legal alcohol purchase age (18 years) [1]. During these years, alcohol features heavily in the lives of many young people as they commence and escalate their drinking behaviours [2, 3]. The process of alcohol initiation and ongoing consumption over these years reflects the embedded role of alcohol in the broader Australian culture and the strong associations made between the ability to tolerate large quantities of alcohol and the successful achievement of adulthood [4, 5]. However, there is some indication that this situation is starting to change. Recent alcohol consumption figures show a substantial increase between 2010 and 2013 in the proportion of adolescents identifying as non-drinkers [3, 6]. This is a highly positive outcome given the association between early alcohol initiation and increased likelihood of risky alcohol consumption behaviours in adulthood [7].

Despite these improvements, those in the 18–24 years age bracket continue to exhibit the highest levels of binge drinking in Australia, with males in this age group being especially likely to drink at harmful levels [3]. This pattern of consumption highlights the need to address the factors associated with young people’s drinking behaviours as they transition between the culturally specified categories of child and adult. The late teens to early adulthood years represent a critical period for interventions because the decisions made about alcohol consumption have implications for individuals’ lifetime use of alcohol [7].

As a particularly alcohol-infused rite of passage ritual in Australia, ‘Schoolies’ represents a phenomenon that can provide insight into the forces that promote heavy drinking in young people during specific events and at specific locations. Schoolies is the period of celebration undertaken by Australian students who have just completed their Year 12 final exams [8, 9]. Many students choose to congregate in various holiday destinations where they achieve geographical and psychological distance from the authority figures who have controlled their lives to date, namely their parents and teachers [9, 10]. Schoolies celebrations can last from a few days to a few weeks and have been traditionally synonymous with the consumption of excessive quantities of alcohol [8, 9, 11–14]. This is despite many participants being 17 years of age, and hence not of legal age to purchase alcohol. This excessive consumption has been found to result in alcohol-related harms for many participants [11, 15]. For example, Lam et al. [15] found that males attending Schoolies at one particular location (Rottnest Island) drank an average of 18 standard drinks per day and females consumed an average of 13 standard drinks per day, resulting in a large majority experiencing adverse outcomes such as hangovers, vomiting, blackouts, and unprotected sex.

The heaviest drinkers during Schoolies tend to be males and those who consume alcohol frequently prior to the event [9, 14–17]. Studies examining Schoolies-related motives and expectancies have found that high levels of intoxication are sought, expected, and obtained by large numbers of participants [9–13, 15, 18, 19]. Consistent with research relating to alcohol consumption by young people elsewhere in similar contexts (e.g., spring break in the US [20, 21]), explicitly-stated motives included opportunities to unwind, socialise with friends, meet new people (especially potential romantic partners), be independent of their parents, and attend music events.

There is growing support for research on event-specific alcohol consumption among young people [20, 22]. In their review of current research relating to harmful alcohol consumption among college students, Mallet et al. [20] recommended that researchers focus on “high-risk windows and activities associated with alcohol consequences”. They noted that such work is critical to inform intervention design and implementation. The present study addresses this need in the context of the Schoolies phenomenon in Western Australia. The aim of the study was to explore expectations of the event and the role of alcohol in students’ anticipated activities and experiences. This study differs from previous work in this area by (i) including a broad range of students who intend to celebrate Schoolies in a variety of ways rather than just focusing on those attending celebrations at one particular geographical location and (ii) actively seeking information relating to any anticipated negative outcomes and perceptions of the consequences of choosing to abstain from alcohol during Schoolies. Insights into the Schoolies phenomenon are likely to be of value in informing strategies to address similar rites of passage such as university orientation events, college drinking parties, and milestone (e.g., 18^th^, 21^st^) birthday celebrations [20, 23–25].

## Methods

An online survey was distributed to Year 11 and 12 students in Western Australia between September and November 2014. The inclusion of Year 11 students permitted analysis of whether expectations differ across the two senior school years. Respondents were recruited via schools and websites: 10 schools from a diverse range of areas across Western Australia were invited to share the online survey link with students and the link was placed on two websites that provided Schoolies information for Western Australian school leavers (e.g., what to expect at different locations and accommodation options).

The online survey included a series of items relating to current alcohol consumption, expected alcohol intake during the Schoolies period, attitudes to Schoolies, and demographic characteristics. Current alcohol consumption levels were assessed by providing a pictorial guide showing standard drink quantities across a broad range of beverage and container types [26] and asking respondents to report the frequency with which they consume alcohol and the average number of standard drinks consumed on each drinking occasion [3]. Intended consumption during Schoolies was assessed with the question, “*On average, how many standard drinks of alcohol per day do you think you will drink during Schoolies?”*. Respondents were also invited to answer several open-ended questions including: *What are you looking forward to most about Schoolies*?, *What are you looking forward to least about Schoolies*?, and *What would be the consequences of not drinking during Schoolies*?.

The demographic and qualitative data were imported into NVivo10 (QSR International) for coding and analysis. Using the constant comparative method [27], the imported content was then coded to a range of conceptual variables that emerged based on the responses provided. Using NVivo’s interrogation functions, text, coding, and matrix searches were used to identify themes in the data. For example, as themes emerged they were compared among different respondent segments using matrix searches to ascertain whether there were any notable differences according to year group, gender, socioeconomic status, or drinking status.

A total of 435 students responded to the survey. Of these, 260 accessed the survey via the link provided to the participating schools and 175 accessed the survey via the Schoolies information websites. Table 1 provides the sample composition by gender, year group, socioeconomic status (calculated using Australian Bureau of Statistics’ socio-economic indexes for areas postcode ratings [28]), current drinking level, and risk level associated with intended number of drinks per day during Schoolies.

**Table 1:** Sample profile (*n* = 435)

Attribute	Number	Percent
Gender		
Male	198	45
Female	237	55
Year group		
Year 11	159	37
Year 12	271	62
Missing	5	1
Socioeconomic status^a^		
Low	50	12
Middle	206	47
High	144	33
Missing	35	8
Current drinking frequency		
≤1/month	233	54
>1/month	122	28
Missing	80	18
Risk level for intended drinks/day during Schoolies		
Nil = no alcohol	88	20
Low risk = 1–4 drinks/day	85	19
High risk = 5+ drinks/day	175	41
Missing	87	20
TOTAL	435	100

^a^As per the Australian Bureau of Statistics’ [28] socio-economic indexes for areas postcode rating

In the results below, the identified themes are illustrated with verbatim extracts that are accompanied by available respondent descriptors using the following code: gender (M, F), socioeconomic status as determined by postcode (low, medium, high), year group (Yr11, Yr12), current drinking in terms of days per month (<=1/month, >1/month), and intended alcohol consumption (nil, low-risk, high-risk). The risk levels assigned to intended daily intake amounts are in accordance with the NHMRC’s [26] definition of high short-term risk of alcohol-related harm being the consumption of more than four standard drinks in a single drinking occasion. Numerous quotes are provided to illustrate coverage across these demographic and consumption variables and to allow the respondents’ voices to be heard while compensating for the inevitably brief nature of qualitative responses to online surveys. Reflecting the nature of data collection, the in-text quotes are provided in the context of the open-ended questions that were posed in the survey.

## Results

### Schoolies as a temporal transition

The responding students appeared highly attuned to role of Schoolies in marking their transition to adulthood. Across their responses, they described the event as a vehicle to move them from their school-based past to a highly anticipated future. The extent to which alcohol appeared to be embedded in the three temporal phases (past, present, and future) of the Schoolies phenomenon presents a range of intervention challenges for those attempting to reduce the high levels of alcohol-related harm among this group. The tables below summarise the key findings in terms of the temporal transitions that were evident in the responses, with exemplar quotes provided. Additional in-text quotes serve to illustrate the extent to which the identified themes were consistent across the various demographic and alcohol use segments.

### The past

For many respondents, Schoolies was an important point of departure for their new lives, both physically and psychologically. They described how it signified leaving behind many core elements of their existing lifestyles and identities, including school attendance, close parental oversight, and, for some, sexual inexperience.

Looking forward to most about Schoolies:

- *Finishing school, entering the adult world, meeting new people and freedom (F, Yr11, low SES, <=1/month, low-risk).*
- *Leaving school, earning new life out of school (M, Yr11, low SES, <=1/month, nil).*
- *Leaving school, going to Uni and becoming free (M, Yr11, high SES < =1/month, high-risk).*

The consumption of alcohol was considered by many to be a key element of the celebration process, as well as being a principal signifier of their new level of maturity. While most respondents would not yet have turned 18 at the time of attending Schoolies, they appeared to consider this event as a defining moment in their transition to adulthood and to consider alcohol as a natural part of this process of leaving school and their childhoods behind: *Enjoying not having school, getting drunk, and forgetting about school* (F, Yr12, high SES, >1/month, high-risk). Table 2 summarises these aspects of Schoolies as a symbolic end to a previous existence.

**Table 2:** Schoolies as a representation of a departure from the past

	Past
Phase characteristics	Leaving behind: - School * School finishing and not having to deal with stupid school problems (M, Yr11, high SES, <=1/month, high-risk).* - Parental oversight * Having a week away from the responsibilities of school and work, as well as parental supervision to just spend time with my friends and just have a fun and memorable experience (F, Yr12, med SES, >1/month, high-risk).* - Sexual inexperience * Enjoy friendships, reminisce on memories, and lose virginity (F, Yr11, med SES, <=1/month, high-risk).*
Role of alcohol	- Marker of moving from past to present * Having awesome drunken times with my best friends as we move on from school and become adults together (M, Yr12, high SES, >1/month, high-risk).* - Symbol of new maturity * Spending time with friends, being independent (away from family), having that sense of ‘growing up’, drinking, partying (F, Yr11, med SES, <=1/month, low-risk).*

### The present

Respondents readily foresaw themselves partaking of the various benefits and activities available to them during the Schoolies period. For the purposes of analysis, this process of imagining themselves during Schoolies is classified as ‘the present’ to provide a demarcation between these findings and those pertaining to the time periods before (‘the past’) and after (‘the future’) Schoolies. As outlined below, the two primary features of respondents’ accounts of their expectations for the period were friends and alcohol.

Social interaction was clearly of great importance, with many mentions of ‘being with friends’ and ‘doing things with friends’. Respondents looked forward to relaxing and drinking together, co-attending major celebration events, and sharing accommodation. Specific activities fell into two major categories – ‘chill’ activities and ‘adrenaline’ activities, which seemed to be looked forward to in equal measure. Chill activities were more passive or sedentary, and included going to the beach, playing games, or sitting around drinking in their accommodation venues. The main adrenaline activity mentioned was ‘partying’, although some expressed an interest in skydiving, scuba diving, and riding rollercoasters. This was the one aspect of the findings that showed a difference according to cohort, with Year 12 students, and especially girls, appearing to have given more thought to their likely experiences during Schoolies.

Looking forward to most about Schoolies:

- *Getting wasted, meeting people, hooking up with people (M, Yr12, med SES).*
- *Have a few drinks and play poker with the lads and some girls (M, Yr12, low SES, <=1/month, high-risk).*
- *Go sky diving and do things extreme (F, Yr12, med SES, low-risk).*
- *Ride a roller coaster (F, Yr12, med SES, <=1/month, high-risk).*

The desire to be with others was not limited to close friends – there was an expectation that social interaction would be enjoyed with a range of other people. However, there were also concerns among those attending celebrations at designated Schoolies locations that it may be difficult to avoid known others whom they disliked and unknown others who were objectionable in some way. Objectionable others included ‘toolies’ (older men who attend Schoolies events in the hope of partnering with young females), drunken revellers unable to control their behaviour, and authority figures such as police who are present in force at these locations.

Looking forward to least about Schoolies:

- *Toolies and the police (F, Yr12, low SES, <=1/month, nil).*
- *Having to avoid cops (M, Yr12, high SES, >1/month, high-risk).*
- *Getting thrown up on or someone spilling a drink on me (F, Yr12, low SES, <=1/month, nil).*
- *People who are stupid drunks and start fights for no reason (M, Yr12, low SES).*

Alcohol had a polarised role in anticipated experiences during Schoolies. As outlined above, it was a fundamental aspect of many activities respondents expected to enjoy, but also featured heavily in descriptions of expected negative experiences. There was acknowledgement that drinking could result in unpleasant consequences for themselves and their peers, including hangovers, needing to assist inebriated friends, receiving unwanted sexual advances, and drink spiking:

Looking forward to least about Schoolies:

- *Hangovers/come downs (M, Yr12, med SES, >1/month, high-risk)*
- *My friends getting really drunk because I will be the one looking after them (F, Yr12, high SES, <=1/month, low-risk).*
- *I am a bit worried about people spiking my drinks (F, Yr12, >1/month, high-risk).*

Alcohol was thus recognised as both a means of achieving satisfying interactions with peers and a potential source of increased vulnerability in relation to undesirable others. These apparently competing aspects of alcohol consumption seemed to be accepted by most respondents as co-existing and inevitable elements of the Schoolies experience. This acceptance was most evident in the responses to the question relating to the perceived consequences of refraining from consuming alcohol during Schoolies. The major anticipated outcome was being socially excluded, which was too high a price for most to contemplate. This concern appeared to be considerably greater among the female respondents.

Consequences of not drinking during Schoolies:

- *People would think I'm boring, I won't fit in, can't party hard (F, Yr12, high SES, >1/month, high-risk).*
- *You’re not fitting, not being like everyone else, it will be harder for you to have a good time when everyone else is drunk and there is no one to talk to (F, Yr12, >1/month, low-risk).*
- *I wouldn't get the ‘desired’ Schoolies experience. I wouldn't be able to relate to those who did consume alcohol (F, Yr11, med SES, <=1/month, nil).*
- *Be left out, no point to going (M, Yr11, high SES, <=1/month, high-risk).*
- *It would make it less of a celebration and more of a normal holiday. Less laughing, less spontaneousness, less strange memories, not as sociable, higher sense of pain - meaning less dancing due to feet hurting, earlier bedtime (F, Yr12, med SES, <=1/month, high-risk).*
- *My friends will probably get mad and think I’m a pussy (M, Yr12, low SES, <=1/month, low-risk).*

Table 3 summarises the role of Schoolies as a symbolic representation of transition to adulthood and the perceived importance of alcohol in facilitating this transition.

**Table 3:** Schoolies as a much-anticipated present

	Present
Phase characteristics	Social interaction: - ‘Being with’ friends * Drink whenever and get a huge house for everyone to stay at (F, Yr12, high SES, >1/month, high-risk).* - ‘Doing with’ friends ○ Chill activities * Chilling out with mates and being able to have casual drinks with people who matter. And also being able to go to the beach every day (F, Yr12, high SES, >1/month, high-risk).* ○ Adrenaline activities * Having a good time and partying hard eg hot biddies (girls) and drinking tons (M, Yr11, low SES, >1/month, high-risk).* - Avoiding undesirable others * Rowdy, drunk men make me really uneasy and I think that there will be a lot of this down at Schoolies. Dealing with them is probably the thing I least am looking forward to (F, Yr12, low SES, <=1/month, low-risk).*
Role of alcohol	- Facilitates interaction with desired others * Stay at a house with a group of friends socialising. Not wild parties, just sitting around listening to music and drinking a bit of alcohol without getting outrageously drunk (F, Yr12, low SES, <=1/month, low-risk).* - Increases susceptibility to undesired others * Rape/assault, drink spiking incidents etc. (F, Yr12, high SES, >1/month, high-risk).* - Negative consequences of heavy consumption expected and tolerated * Dealing with hangovers and looking after drunk mates (M, Yr12, high SES, >1/month, high-risk).*

### The future

As well as valuing Schoolies for signifying the end of their school lives and constituting a fun experience, respondents perceived the event and its consequences as being important to their future lives. Life after Schoolies was understood to include lasting effects from the time they spent celebrating with their peers, including memories that could be shared with others, the consolidation of existing friendships, and the commencement of new friendships.

Looking forward to most about Schoolies:

- *Partying, meeting new people, making memories (M, Yr12, >1/month, high-risk).*
- *Simply have quality bonding time with close friends (M, Yr11, med SES, >1/month).*
- *Spending those final moments with my close friends and being in the atmosphere as well as meeting new people (F, Yr12, med SES).*

In particular, alcohol was recognised as a disinhibitor that permits the occurrence of unusual and amusing events with their peers, which in turn become the substance of memories to be enjoyed at later times. To refrain from drinking was therefore associated with a future devoid of these memories: *Won’t have as many funny memories to look back on (M, Yr12, high SES, <=1/month, high-risk).* Table 4 provides an overview of the forward-looking aspect of Schoolies and the perceived role of alcohol in assisting the progression to a desirable future.

**Table 4:** Schoolies as symbolic of the future

	Future
Phase characteristics	Consequences: - Memories to share * Leaving school and making memories (M, Yr11, high SES, <=1/month, nil).* - Solidified friendships * Hope I can strengthen my relationships with those I’m close to (M, Yr11, high SES, <=1/month, low-risk).* - New relationships * Meeting new people and partying for a week with the people I love (F, Yr12, high SES, >1/month, high-risk).*
Role of alcohol	- Permits the occurrence of memorable events through disinhibition * (If I didn’t drink I) wouldn't have fun with my friends and wouldn’t get the whole Schoolies experience, no good stories to tell (F, Yr12, high SES, >1/month, high-risk).* - Facilitates relationship maintenance and development * (If I don’t drink) I won't fit into the group and I'll become isolated from the group (M, Yr12, med SES, <=1/month, nil).*

## Discussion

As shown in Table 5, various conceptual constructs suggested by the findings provide insight into possible approaches to harm reduction. The extent to which the identified themes were consistent across demographic segments was notable, but perhaps unsurprising given the similar exposure students have to Schoolies-related media attention and their immersion in school environments where their peers would readily share thoughts and feelings relating to their Schoolies expectations, potentially resulting in high levels of attitudinal convergence. The commonalities in responses are useful in permitting more universal harm prevention strategies to be implemented than would be possible if highly disparate themes were identified between groups.

**Table 5:** Relevant conceptual frameworks and intervention implications

	Past	Present	Future
Conceptual frameworks	- Rite of passage rituals - Maturation theories - Social learning theory	- In-groups and out-groups - Self-efficacy - Regulatory focus	- Social memories - Social capital
Intervention implications	- Reduce the role of alcohol in the rite of passage to adulthood	- Increase availability of, access to, and awareness of alternative activity options - Improve self-efficacy relating to moderate drinking and refusal skills	- Work with young people to develop and deliver alternative activities

Schoolies’ links with the past reinforce previous work highlighting (i) the role of alcohol in the rite of passage to adulthood [29] and (ii) the maturation processes involved in this transition [30]. These processes indicate the need for change at a cultural level to reduce the association between heavy alcohol consumption and coming of age. This is a challenging task in a context such as Australia where alcohol is an acknowledged element of the social fabric [4, 31]. However, there are indications that current alcohol control policies are having some effect at a population level, with recent reductions in the proportion of Australians who drink, the frequency with which drinking occurs, and the proportion who engage in risky drinking [3]. These reductions are especially evident among teenagers and young adults, although almost half of those aged 18–24 continue to drink at levels associated with short-term harm at least monthly [3]. Further decreases in consumption among underage drinkers may be expected as the proportion of those modelling harmful behaviours is reduced (as per social learning theory [32]).

The task at hand is to translate these general societal improvements in risky drinking to Schoolies. Part of this process is likely to be the provision of alternative Schoolies activities that are appealing to the target group while being inconsistent with the excessive consumption of alcohol [8, 10]. The present findings suggest that to be attractive to school leavers, such activities would need to be highly conducive to social interaction and largely devoid of interference from authority figures (predominantly parents and police), while still being capable of expressing the idea of ‘shared emancipation’ [33].

In terms of respondents’ anticipated experiences during Schoolies, there were clear demarcations expressed between in-groups and out-groups [34]. In-group connections were so strong that the consumption of alcohol was generally perceived to be necessary because of its role in facilitating social interactions with valued others during their celebrations. This reflects the outcomes of previous research that has emphasised the importance of substance use in building and maintaining friendship networks within schools [35]. Efforts to reduce excessive consumption would therefore need to (i) acknowledge and account for these assumptions and ensure any interventions address perceived social norms and (ii) provide strategies that would assist individuals to reframe their expectations of themselves and their peers. The identified aversion to specific out-groups may assist in this process. Girls in particular may be receptive to messages about minimising their alcohol intake to reduce their risk of harm. Increasing their self-efficacy (e.g., by actively planning ahead) to moderate their drinking and refuse unwanted drinks may assist in re-defining the perceived trade-off between physical well-being and social acceptance [36].

The desire of respondents to take elements of their Schoolies’ experiences into their futures further illustrates the transitional nature of the event. This desire was manifest in a strong intention to create shared memories with their peers that could be relived in the future. Theories of social remembering [37] emphasise the role of memory sharing among young people as a mechanism via which both these memories and the relationships they signify can persist across the transition from adolescent to adulthood. A further future-oriented intention related to the establishment of new friendships during Schoolies, which may be interpreted as an effort to enhance their social capital through expanding their social networks and making connections that could be beneficial in the future [38]. Interventions designed to either reduce alcohol consumption during existing Schoolies events or redirect participants to other activities thus need to be cognisant of the need to provide opportunities for memory-making and the development of new social connections. An element of this approach may be to highlight the negative consequences of forming unpleasant memories and the potential to create damaging impressions among others who may be encountered later in other places, such as university or in the workplace, or who may be potential future romantic partners.

Overall, the findings of the present study indicate that Schoolies constitutes a ‘turning point event’. As defined by Rutter [30], turning point events are characterised by (i) a marked change in environment or situation that has developmental consequences and (ii) persistent effects over time. Respondents’ expectations of their Schoolies’ experiences reflected both these backward- and forward-looking elements, demonstrating their understanding that this particular event represents a major transition from one state of being to another and has the potential to influence their future lives. Current interventions typically seek to limit school leavers’ activities via alcohol control strategies and regulations and to sensitise them to the need to focus on their immediate safety in the celebration locations. While there is likely to be an ongoing need for these kinds of approaches, the present findings provide additional insights into why such strategies are unlikely to be effective in isolation and highlight the need to consider alternative approaches [31].

The concept of regulatory focus may be useful in this regard [39]. Most of the study participants exhibited a ‘promotion’ regulatory focus in that they were intent on maximising the fun and enjoyment to be had during Schoolies. Relatively few exhibited a ‘prevention’ regulatory focus characterised by a perceived need to protect their well-being. This was evidenced by frequent mentions of the intention to drink heavily, the anticipation of hangovers and other negative outcomes, and the extensive comments made about the extent to which they would miss out on the fun of Schoolies should they elect not to consume alcohol. Some existing Schoolies interventions attempt the herculean task of converting individuals from a promotion focus to a prevention focus by providing education relating to the dangers associated with excessive alcohol consumption [14, 40]. An alternative or complementary approach would be to work with this group to identify the other celebratory options that could supply opportunities for memory-building and the strengthening and building of friendships, but that are not closely associated with excessive alcohol consumption. This approach would provide behavioural alternatives rather than being perceived as limiting the new freedoms that are so highly sought, and is consistent with work highlighting the need for primary stakeholder involvement in the development of alcohol harm-reduction strategies [41].

### Limitations and future research

The main limitation of the present study was the use of qualitative data generated via an online survey. This approach prevented the ability to probe for further clarification as can be done with other forms of qualitative data collection. It also resulted in relatively brief responses. However, these limitations were at least partially offset by the large sample size and consistency in responses, indicating that the themes identified are likely to be robust and therefore worthy of further investigation. A second limitation was the inability to calculate response rates due to the unknown number of students who were exposed to the survey invitation at school or online, although the diversity of the sample provides some assurance of the range of Western Australian students who participated in the study.

A further limitation relates to the context of the study. Data collection was limited to one Australian state, and the young people involved in this study had a younger average age relative to much of the rest of the country due to different school age intake requirements in Western Australia. This highlights the need for national replication studies to assess the extent to which the identified themes relate to Schoolies in other states. Finally, the self-reported nature of the data relating to existing and anticipated alcohol intake levels constitutes a limitation given the propensity for drinkers to incorrectly estimate or represent their intake [42, 43]. However, this limitation is shared by most other studies of alcohol-related attitudes and behaviours due to the private nature of much alcohol consumption. Future research could partially address this limitation by observing Schoolies during their celebrations and estimating their intake, although this would limit data collection to publicly occurring celebrations, which would only capture one aspect of Schoolies celebrations.

## Conclusions

The data generated in this study illustrate that rather than being a discrete event that occurs at a single point in time at particular locations, Schoolies has tendrils that reach into the past and future. As such, interventions that treat Schoolies as an isolated, annual event that can be managed by focusing on specific groups of young people in specific places at a particular point in time may fail to adequately address the complexity of the forces at play. Instead, the findings suggest a need to appreciate the extent to which the event constitutes a social and psychological phenomenon that transcends time and place for those involved.

## Abbreviations

F, female; M, male; Med, medium; SES, socioeconomic status
